# Eicosanoids in inflammation in the blood and the vessel

**DOI:** 10.3389/fphar.2022.997403

**Published:** 2022-09-27

**Authors:** Adriana Yamaguchi, Eliana Botta, Michael Holinstat

**Affiliations:** ^1^ Department of Pharmacology, University of Michigan Medical School, Ann Arbor, MI, United States; ^2^ Department of Internal Medicine, Division of Cardiovascular Medicine, University of Michigan Medical School, Ann Arbor, MI, United States

**Keywords:** eicosanoids, inflammation, oxygenases, blood, blood vessel

## Abstract

Polyunsaturated fatty acids (PUFAs) are structural components of membrane phospholipids in cells. PUFAs regulate cellular function through the formation of derived lipid mediators termed eicosanoids. The oxygenation of 20-carbon PUFAs via the oxygenases cyclooxygenases, lipoxygenases, or cytochrome P450, generates a class of classical eicosanoids including prostaglandins, thromboxanes and leukotrienes, and also the more recently identified hydroxy-, hydroperoxy-, epoxy- and oxo-eicosanoids, and the specialized pro-resolving (lipid) mediators. These eicosanoids play a critical role in the regulation of inflammation in the blood and the vessel. While arachidonic acid-derived eicosanoids are extensively studied due to their pro-inflammatory effects and therefore involvement in the pathogenesis of inflammatory diseases such as atherosclerosis, diabetes mellitus, hypertension, and the coronavirus disease 2019; in recent years, several eicosanoids have been reported to attenuate exacerbated inflammatory responses and participate in the resolution of inflammation. This review focused on elucidating the biosynthesis and the mechanistic signaling of eicosanoids in inflammation, as well as the pro-inflammatory and anti-inflammatory effects of these eicosanoids in the blood and the vascular wall.

## 1 Introduction

Eicosanoids are a family of fatty acid metabolites generated from 20-carbon polyunsaturated fatty acids (PUFAs) synthesized by enzymatic oxygenation pathways involving a distinct family of enzymes, the oxygenases (Khanapure et al., 2007). Eicosanoids are not stored, but promptly synthesized *de novo* after cell activation (Bozza et al., 2011) through a highly regulated event, primarily involving three oxygenases: cyclooxygenases (COXs), P450 cytochrome epoxygenases (CYP450), and lipoxygenases (LOXs) (Alvarez and Lorenzetti, 2021). The formed eicosanoids function to regulate a physiological response, including tissue homeostasis, pain, host defense, and inflammation (Esser-von Bieren, 2019). Due to the observed critical role of eicosanoids in physiological and pathological inflammation, they have been implicated in the pathogenesis of major diseases including cardiovascular disease, diabetes mellitus, hypertension, and more recently, the coronavirus disease 2019 (COVID-19) (Wang and Dubois, 2010; Fava and Bonafini, 2018; Hammock et al., 2020; Bosma et al., 2022). Although eicosanoids are usually associated with pro-inflammatory responses (Aoki and Narumiya, 2012), they are also known to play a key role in reducing inflammation by promoting the resolution of inflammation (Pan et al., 2022), limiting immune cell infiltration, and initiating tissue repair mechanisms (Serhan and Levy, 2018; Díaz Del Campo et al., 2022). This review focuses on the role of eicosanoids on inflammation in the blood and the vascular wall and discusses key discoveries related to the regulatory mechanism of these lipid mediators in inflammation in the blood vessel.

## 2 Eicosanoid biosynthesis

**FIGURE 1:**
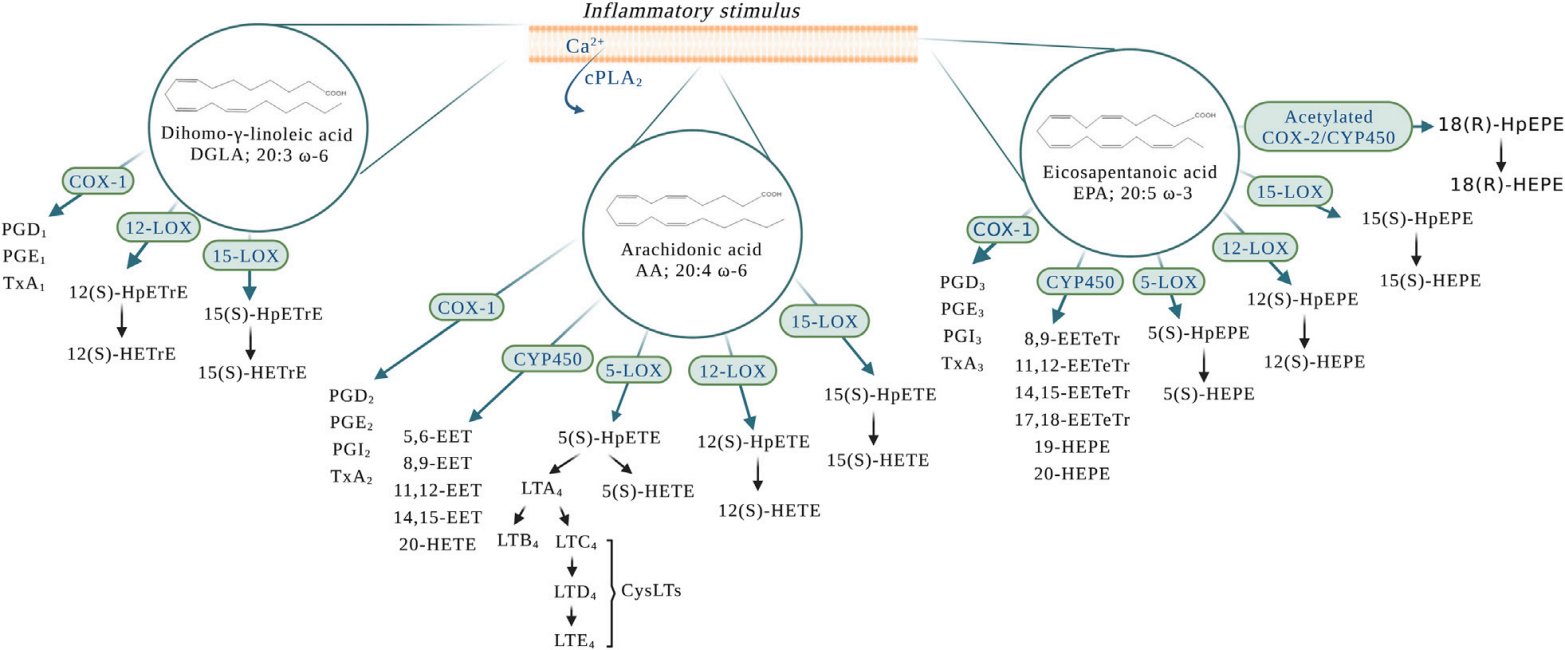
Eicosanoid biosynthesis in the blood and the vessel upon an inflammatory stimulus. *AA*: arachidonic acid; *COX-1*: cyclooxygenase-1; *COX-2*: cyclooxygenase-2; *cPLA*_*2*_: cytoplasmic phospholipase A_2_; *CYP450:* P450 cytochrome epoxygenase; *CysLTs:* cysteinyl leukotrienes; *DGLA*: dihomo-γ-linolenic acid; *EPA*: eicosapentaenoic acid; *LTA*_*4*_: leukotriene A_4_; *LTB*_*4*_: leukotriene B_4_; *LTC*_*4*_: leukotriene C_4_; *LTD*_*4*_: leukotriene D_4_; *LTE*_*4*_: leukotriene E_4_; *PGD*_*1*_: prostaglandin D_1_; *PGD*_*2*_: prostaglandin D_2_; *PGD*_*3*_: prostaglandin D_3_; *PGE*_*1*_: prostaglandin E_1_; *PGE*_*2*_: prostaglandin E_2_; *PGE*_*3*_: prostaglandin E_3_; *PGI*_*2*_ *:* prostaglandin I_2_ or prostacyclin; *PGI*_*3*_ *:* prostaglandin I_3_; *TxA*_*1*_: thromboxane A_1_; *TxA*_*2*_: thromboxane A_2_; *TxA*_*3*_: thromboxane A_3_; *5(S)-HEPE*: 5(S)-hydroxyeicosapentaenoic acid; *5(S)-HpEPE*: 5(S)-hydroperoxyeicosapentaenoic acid; *5(S)-HETE: 5*(S)-hydroxyeicosatetraenoic acid; *5(S)-HpETE: 5*(S)-hydroperoxyeicosatetraenoic acid; *5-LOX:* 5-lipoxygenase; *5,6-EET*: 5,6-epoxyeicosatrienoic acid; *8,9-EET*: 8,9-epoxyeicosatrienoic acid; *8,9-EETeTr*: 8,9-epoxyeicosatetraenoic acid; *11,12-EET*: 11,12-epoxyeicosatrienoic acid; *11,12-EETeTr*: 11,12-epoxyeicosatetraenoic acid; *12(S)-HETE: 12*(S)-hydroxyeicosatetraenoic acid; *12(S)-HpETE: 12*(S)-hydroperoxyeicosatetraenoic acid; *12(S)-HETE: 12*(S)-hydroxyeicosatetraenoic acid; *12(S)-HpETE: 12*(S)-hydroperoxyeicosatetraenoic acid; *12(S)-HETrE:* 12(S)-hydroxyeicosatrienoic acid; *12(S)-HpETrE:* 12(S)-hydroperoxyeicosatrienoic acid; *12-LOX:* 12-lipoxygenase; *14,15-EET*: 14,15-epoxyeicosatrienoic acid; *14,15-EETeTr*: 14,15-epoxyeicosatetraenoic acid; *15-LOX:* 15-lipoxygenase; *15(S)-HETE: 15*(S)-hydroxyeicosatetraenoic acid; *15(S)-HpETE: 15*(S)-hydroperoxyeicosatetraenoic acid; *15(S)-HETE: 15*(S)-hydroxyeicosatetraenoic acid; *15(S)-HpETE: 15*(S)-hydroperoxyeicosatetraenoic acid; *15(S)-HETrE:* 15(S)-hydroxyeicosatrienoic acid; *15(S)-HpETrE:* 15(S)-hydroperoxyeicosatrienoic acid; *17,18-EETeTr*: 17,18-epoxyeicosatetraenoic acid; *18(R)HpEPE:* 18(R)-hydroperoxyeicosapentaenoic acid; *18(R)-HEPE:* 18(R)-hydroxyeicosapentaenoic acid; *19-HEPE*: 19-hydroxyeicosapentaenoic acid; *20-HEPE*: 20-hydroxyeicosapentaenoic acid; *20-HETE*: 20-hydroxyeicosatetraenoic acid.

**FIGURE 2:**
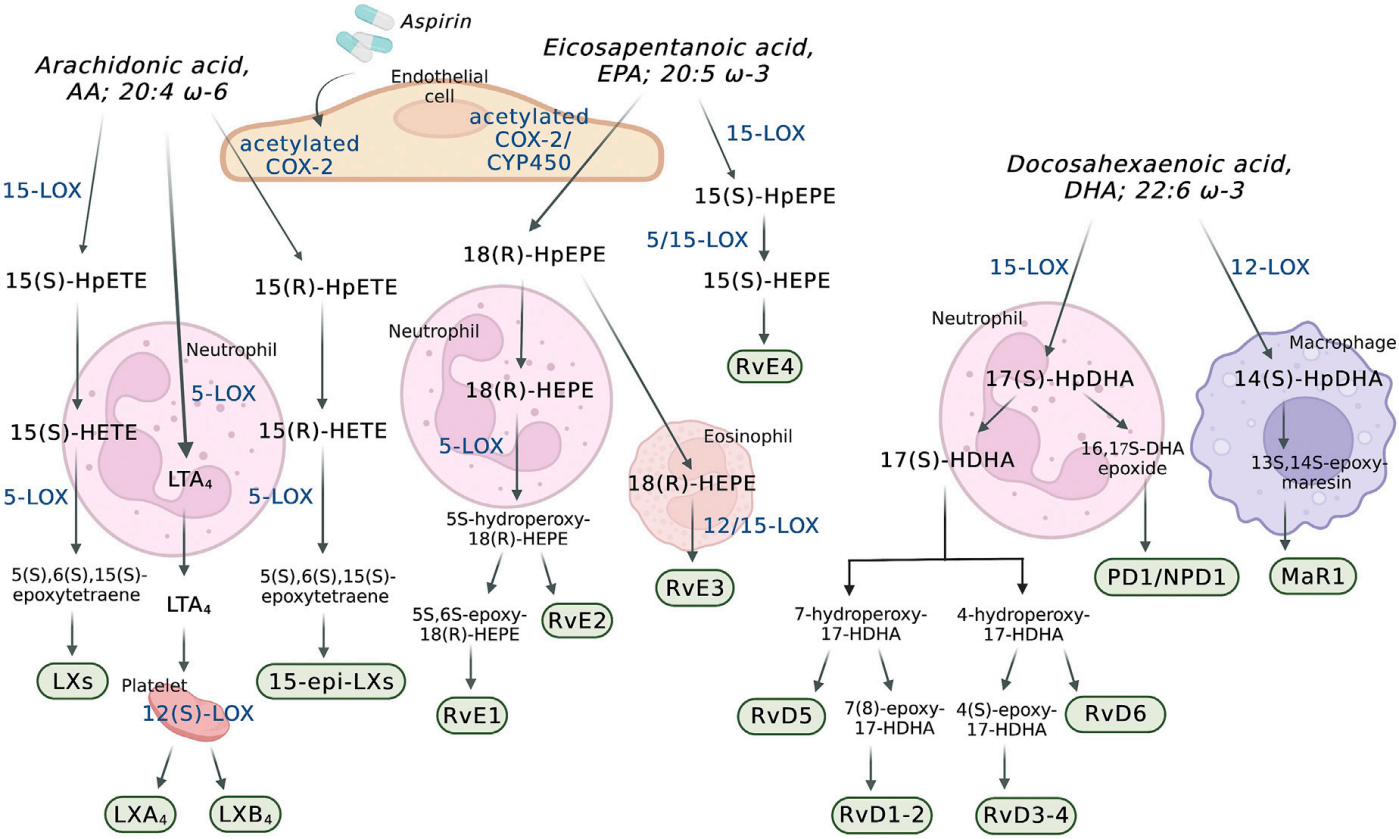
Transcellular biosynthesis of eicosanoids in the blood and the vascular wall. *AA*: arachidonic acid; *COX-2:* cyclooxygenase-2; *CYP450:* P450 cytochrome epoxygenase ***;*** *DHA:* docosahexaenoic acid*; EPA*: eicosapentaenoic acid; *LTA*_*4*_: leukotriene A_4_; *LXs:* lipoxins; *LXA*_*4*_ *:* lipoxin A_4_; *LXB*_*4*_ *:* lipoxin B_4_; *MaR1:* maresin 1; *PD1/NPD1:* protectin D1/neuroprotectin D1; RvD1-2: resolvin D1-2; RvD3-4: resolvin D3-4; RvD5: resolvin D5; RvD6: resolvin D6; *RvE1:* resolvin E1; *RvE2:* resolvin E2;* RvE3:* resolvin E3; *RvE4:* resolvin E4; *5-LOX:* 5-lipoxygenase; *12(S)-LOX:* 12(S)-lipoxygenase*; 14(S)-HpDHA:* 14(S)-hydroperoxydocosahexaenoic acid*; 15-LOX:* 15-lipoxygenase; *15(S)-HEPE*: 15(S)-hydroxyeicosapentaenoic acid; *15(S)-HpEPE*: 15(S)-hydroperoxyeicosapentaenoic acid; *15(S)-HETE: 15*(S)-hydroxyeicosatetraenoic acid; *15(S)-HpETE: 15*(S)-hydroperoxyeicosatetraenoic acid; *15-epi-LX*_*s*_ *:* 15-epi-lipoxins; *17(S)-HDHA:* 17(S)-hydroxydocosahexaenoic acid; *17(S)-HpDHA:* 17(S)-hydroperoxydocosahexaenoic acid; *18(R)-HEPE*: 18(R)-hydroxyeicosapentaenoic acid; *18(R)-HpEPE*: 18(R)-hydroperoxyeicosapentaenoic acid.

The superclass of eicosanoids expressed in the blood includes classical eicosanoids, prostaglandins (PGs), leukotrienes (LTs), and thromboxanes (Txs), and the more recently discovered, specialized pro-resolving (lipid) mediators (SPMs), as well as hydroxy-, hydroperoxy-, epoxy-, and oxoeicosanoids (Fahy et al., 2009). The SPMs include lipoxins (LXs), resolvins, protectins (PD), their aspirin-triggered (AT) isomers, and maresins (MaR) (Chiang and Serhan, 2020). One of the best-studied classes of these lipid mediators are the eicosanoids derived from the 20-carbon PUFAs such as eicosapentaenoic acid (EPA; 20:5 ω-3), dihomo-γ-linolenic acid (DGLA; 20:3 ω-6), and arachidonic acid (AA; 20:4 ω-6) (Astarita et al., 2015) (Figure 1), with the last being the most abundant PUFA in the phospholipid of human cell membranes (Sonnweber et al., 2018). Regarding the newly discovered SPMs, LXs are generated from AA, E-series resolvins (RvEs) are synthesized from EPA, and D-series resolvins (RvDs), PDs, and MaRs are formed from docosahexaenoic acid (DHA; 22:6 ω-3) (Calder, 2020a; Chiang and Serhan, 2020) (Figure 2). The initial event of eicosanoid biosynthesis consists of cellular activation which leads to an increased influx of calcium; and subsequently, the translocation of cytoplasmic phospholipase A_2_ (cPLA_2_) to the membrane resulting in the cleavage of the PUFA from the *sn*-2 position of the glycerophospholipid (Dennis et al., 2011) to be further oxygenated by respective enzymes. Eicosanoid biosynthesis differs between the type of PUFA being oxidized and the enzymes metabolizing those PUFAs. The freed PUFAs can be oxygenated by several enzymes including COXs, LOXs and CYP450s (Hajeyah et al., 2020) (Figure 1). An alternative biosynthesis pathway forming the AT-isomers can be triggered by aspirin. For example, in the presence of aspirin, AA and EPA form 15(R)-HpETE and 18(R)-HpEPE, respectively (Calder, 2020a) (Figure 2).

### 2.1 The cyclooxygenase-dependent synthesis

Cyclooxygenases are a widely distributed enzyme in mammalian tissues and exist in two isoforms, COX-1 and COX-2 (Vane et al., 1998). The activation of COX leads to the generation of PGs and Txs, which are collectively named as prostanoids. COX oxygenates AA into series 2 PGs (PGD_2_, PGE_2_, PGI_2_, and TxA_2_) (Figure 1). Series 1 PGs (PGD_1_, PGE_1_, and TxA_1_) and series 3 PGs (PGD_3_, PGE_3_, PGI_3_, and TxA_3_,) are produced from the oxygenation of DGLA and EPA, respectively (Lagarde et al., 2013; Sergeant et al., 2016) (Figure 1).

In patients taking aspirin, COX-2 is involved in the formation of LXs and RvEs through transcellular biosynthesis (Figure 2). In endothelial cells, aspirin causes an irreversible acetylation of COX-2, which oxygenates AA to form 15(R)-hydroxyeicosatetraenoic acid (15(R)-HETE) and EPA to form 18(R)-hydroxyeicosapentaenoic acid (18(R)-HEPE). While 15(R)-HETE is further used by adherent leukocyte and other endothelial cells to form the 15-epimeric-LXs (15-epi-LXs) (Fu et al., 2020), 18(R)-HEPE is further metabolized into RvE1 and RvE2. It is important to mention that 18(R)-HEPE can also be formed through oxygenation of EPA by CYP450s (Serhan and Petasis, 2011).

### 2.2 The lipoxygenase-dependent synthesis

Lipoxygenases are a family of nonheme iron-containing enzymes (Kuhn et al., 2015) which are categorized accordingly to their positional specificity of AA oxygenation: 5-LOX, 12-LOX, and 15-LOX. LOX isozymes are further characterized by tissue expression and stereospecificity (S or R), such as the platelet-type 12-(S)-LOX and the epithelial 12-(R)-LOX (Brash, 1999), as an example. Regarding the expression of LOXs in the blood, 12(S)-LOX is only expressed in platelets and 15-LOX-1 is expressed in eosinophils, monocytes, macrophages, and reticulocytes (Jiang et al., 2006). The expression of 5-LOX is found in myeloid cells including neutrophils, macrophages, monocytes and basophils (Yeung et al., 2017).

The LOXs are able to oxygenate AA to form hydroperoxyeicosatetraenoic acids (HpETEs) (Figure 1), which are rapidly converted to hydroxy derivative HETEs in the blood (Tourdot and Holinstat, 2017). In a similar manner, the LOX-derived eicosanoids from DGLA and EPA are converted to hydroperoxyeicosatrienoic acids (HpETrEs) and hydroperoxyeicosapentaenoic acids (HpEPEs), which are further hydrolyzed to hydroxyeicosatrienoic acids (HETrEs) and hydroxyeicosapentaenoic acids (HEPEs), respectively (Yeung et al., 2017). 5-LOX is best known for its ability to produce LTs (Figure 1). The oxygenation of AA by 5-LOX generates 5(S)-HpETE, which is further converted to the unstable leukotriene A_4_ (LTA_4_). This intermediate eicosanoid is either converted to the leukotriene B_4_ (LTB_4_) or leukotriene C4 (LTC_4_) in cells that possess LTC_4_ synthase activity, such as platelets and endothelial cells, and sequential degradation of the LTC_4_ by peptidases forms LTD_4_ and LTE_4_ (Figure 1). These three products, LTC_4_, LTD_4_, and LTE_4_, are collectively named cysteinyl LTs (cysLTs). The production of cysLTs appears to be restricted to leukocytes, including eosinophils, basophils, and macrophages. However, under inflammatory stimulus, transcellular activity can result in cysLTs formation in endothelial cells (Feinmark and Cannon, 1986). This mechanism favors cells unable to produce LTA_4_, such as vascular endothelial cells, platelets and blood peripheral monocytes, to use LTA_4_ generated from surrounding cells (such as leukocytes) to produce LTC_4_ and the other cysLTs (Colazzo et al., 2017). LTA_4_ can also be used by other cells in the blood to form the LXs. Lipoxins A (LXA_4_) and B (LXB_4_) are formed through a transcellular mechanism between polymorphonuclear leukocytes (PMNs) (5-LOX) and platelets (12(S)-LOX) (Recchiuti and Serhan, 2012). In addition to the transcellular mechanism, lipoxins are synthesized from AA via 15-LOX in neutrophils and monocytes. In these cells, AA is converted to 15(S)-hydroperoxyeicosatetraenoic acid (15(S)-HpETE), which is subsequently converted to lipoxins A and B (Chandrasekharan and Sharma-Walia, 2015) (Figure 2).

The LOXs are also involved in the biosynthesis of others SPMs derived from DHA and EPA. The D-series resolvins 1–6 (RvD1-RvD6) are SPMs derived from the DHA-derived 17(S)-hydroperoxydocosahexaenoic acid (17(S)-HpDHA), which is synthesized through the oxygenation of DHA by 5-LOX in PMNs and macrophages (Serhan and Levy, 2018) (Figure 2). DHA is also a precursor for the maresins (MaR) and protectins. Maresin 1 (MaR1) is generated from the precursor DHA-derived 14(S)-hydroperoxydocosahexaenoic acid (14(S)-HpDHA) and its biosynthesis was first described in human macrophages via 12-LOX-mediated biosynthesis (Serhan et al., 2009). MaR1 is also synthesized during platelet-PMN interactions (Serhan and Levy, 2018). In leukocytes, the biosynthesis of the protectin D1/neuroprotectin D1 (PD1/NPD1) has the DHA-derived 17(S)-HpDHA as the intermediate precursor and it occurs via a 15-LOX-mediated pathway (Serhan et al., 2015; Chiang and Serhan, 2020) (Figure 2). Also in leukocytes, aspirin triggers the biosynthesis of the DHA-derived aspirin-triggered neuroprotection D1/protectin D1 [AT-(NPD1/PD1)] (Serhan et al., 2011a; Serhan et al., 2015). The E-series resolvins 1–4 (RvE1-4) are generated from a common precursor, the EPA-derived 18(R)-hydroxyeicosapentaenoic acid (18(R)-HEPE) (Figure 2). RvE1 and RvE2 are synthesized by PMNs via the 5-LOX pathway, whereas RvE3 is synthesized by eosinophils via the 12/15-LOX pathways (Serhan and Petasis, 2011; Isobe et al., 2012). Currently, the synthesis of RvE4 has only been shown in *in vitro* studies using purified recombinant human 5-LOX and 15-LOX (Serhan and Petasis, 2011; Libreros et al., 2020).

### 2.3 The cytochrome P450-dependent synthesis

CYP450s belong to a family of heme-containing monooxygenases (Cook et al., 2016) that are known for their role in the metabolism of eicosanoids from PUFAs (Zhao et al., 2021). CYP epoxygenase metabolizes AA into epoxyeicosatrienoic acids (EETs) (Figure 1). Four regioisomeric cis-epoxyeicosatrienoic acids have been described: 5.6-, 8.9-, 11.12-, and 14,15-EET. Upon hydration by soluble epoxide hydrolase (sEH), EETs are rapidly converted to more stable and less biologically active metabolites, dihydroxyeicosatrienoic acids (DHETs) (Spiecker and Liao, 2005). Additionally, members of the CYP4A and CYP4F subfamilies also oxygenate AA to produce 20-hydroxyeicosatetraenoic acid (20-HETE) (Arnold et al., 2010) (Figure 1), which undergoes additional oxidation to 20-hydroxy-prostaglandin G_2_ and H_2_ (Schwartzman et al., 1989; Kaduce et al., 2004; Hoxha and Zappacosta, 2020). EPA can also be a substrate for CYP450 catalysis. The major CYP450-dependent metabolites derived from EPA include epoxyeicosatetraenoic acids (EETeTrs, 5.6-, 8.9-, 11.12-, and 14,15-EETeTrs), 19- and 20-hydroxyeicosapentaenoic acids (19- and 20-HEPE) (Figure 1).

## 3 Eicosanoid mode of action

### 3.1 Prostanoid receptors

PGs exert their biological effects in the blood in an autocrine and paracrine manner by activating their respective cell surface G protein-coupled receptors (GPCRs) (Ricciotti and FitzGerald, 2011; Biringer, 2021). There are at least eight known prostanoid receptor subfamilies in the blood and the vascular wall (Funk, 2001) (Table 1). Four of the receptor subtypes bind PGE_2_, E prostanoid receptor (EP) 1, EP2, EP3, and EP4 in platelets and vascular smooth muscle cells (VSMCs) and two bind PGD_2_ (DP1 and DP2) (Aoki and Narumiya, 2012). While PGF_2α_ binds to FP, the PGI_2_ and TxA_2_ receptors are known as IP and TP, respectively (Wang and Dubois, 2010). The IP is expressed in the endothelium, VSMCs and platelets. There are two isoforms of human TP (TP_α_, TP_β_) in platelets, vascular smooth muscle cells, and macrophages, and FP (FP_A_, FP_B_) in VSMCs (Ricciotti and FitzGerald, 2011; Gilroy and Bishop-Bailey, 2019). DP2 is also known as a chemoattractant receptor-homologous molecule (CRTH/DP2) expressed in T helper 2 cells, that responds to PGD_2_ but belongs to the family of chemokine receptors (Ricciotti and FitzGerald, 2011; Aoki and Narumiya, 2012).

**TABLE 1:** The signal transduction of the eicosanoid receptors.

Eicosanoid	Receptor Subtype	G-protein coupled	Intracellular signaling
**PGE**_**2**_	EP1	G_αq_	↑ IP_3_, ↑ Ca^2+^
	EP2	G_αs_	AC activation, ↑ cAMP, PKA activation
	EP3	G_αi_ or G_α12_	↑ Ca^2+^, Rho activation
	EP4	G_αs_	AC activation, ↑ cAMP, PKA activation
**PGD**_**2**_	DP1	G_αs_	AC activation, ↑ cAMP, PKA activation
	DP2 (CRTH/DP2)	G_αi_	↓ cAMP, ↑ Ca^2+^
**PGF**_**2**α_	FP_A_, FP_B_	G_αq_	↑ IP_3_, ↑ Ca^2+^
		G_α12/13_	Rho activation
**PGI**_**2**_	IP	G_αs_	AC activation, ↑ cAMP, PKA activation
**TxA**_**2**_	TP_α_, TP_β_	G_αq_	↑ IP_3_, ↑ Ca^2+^
		G_α12/13_	Rho activation
**LTB**_**4**_	BLT_1_	G_αi_	↑ Ca^2+^
**LTB**_**4**_ **, 12-HHT, HETEs**	BLT_2_	G_αi_	Phosphorylation of MAPKs and PI3K/Akt, NF-kB activation
**LTC**_**4**_ **, LTD**_**4**_	CysLT_1_	G_αi/o_	PLCβ activation, ↑ Ca^2+^, ERK phosphorylation
	CysLT_2_	G_αq/11_	PLCβ activation, ↑ IP_3_, ↑ Ca^2+^
**EETs**	PPARγ	-	NF-κB inhibition, STAT3 activation
**12(S)-HETrE**	IP	G_αs_	AC activation, ↑ cAMP, PKA activation
**12(S)-HETE**	GPR31	G_αi_	AC inhibition, Rap1 and p38 activation
**20-HETE**	GPR75	G_αq/11_	IP_3_, ↑ Ca^2+^, activation of Rho kinase, NF-κB and MAPK/ERK pathway
**11(S)-HpDPA**_ω**-6**_, **14(S)-HpDPA**_ω**-6**_	PPARα	-	PKC inhibition, ↓ Ca^2+^
**RvE1**	BLT_1_	ND	Phosphorylation of rS6
	**ERV1**/ChemR23	ND	Phosphorylation of Akt and rS6
**RvD1, LXs**	ALX/FPR2, **GPR32**	ND	ND
**RvD2**	GPR18	ND	↑ cAMP, ↑ CREB and STAT3 phosphorylation
**RvD5**	GPR32	ND	↓ Expression of NF-kB
**MaR1**	LGR6	ND	↑ CREB and ERK phosphorylation, NF-κB inhibition
**PD1/NPD1**	GPR37	ND	↑ Ca^**2+**^

Note: *AC:* adenylyl cyclase; *Akt:* protein kinase B; *BLT:* leukotriene B_4_ receptor; *cAMP:* cyclic adenosine monophosphate; *ALX/FPR2:* formyl peptide receptor 2; *ChemR23:* chemokine-like receptor 1; *CREB:* cAMP-response element binding protein; *CRTH:* chemoattractant receptor-homologous molecule; *CysLT:* cysteinyl leukotriene receptor*; DP:* prostaglandin D receptor; *EETs:* epoxyeicosatrienoic acids*; EP:* E prostanoid receptor; *ERK:* extracellular signal-regulated kinase; *ERV1:* resolvin E1 receptor; *FP:* prostaglandin F receptor; *GPR18:* G-protein coupled receptor 18; *GPR31:* G protein-coupled receptor 31; *GPR32:* G protein-coupled receptor 32; *GPR37:* G protein-coupled receptor 37; *GPR75:* G protein-coupled receptor 75; *HETE:* hydroxyeicosatetraenoic; *IP:* prostacyclin receptor; *IP*_*3*_ *:* inositol triphosphate; *LGR6:* leucine-rich repeat-containing G protein-coupled receptor 6; *LTB*_*4*_ *:* leukotriene B_4_; *LTC*_*4*_ *:* leukotriene C_4_; *LTD*_*4*_ *:* leukotriene D_4_; *LXs:* lipoxins; *MAPK:* mitogen-activated protein kinase; *MaR1:* maresin 1; *ND:* non-determined in cells in the blood or the vessel; *NF-κB:* nuclear factor kappa B; *PD1/NPD1:* protectin D1/neuroprotectin D1; *PGD*_*2*_ *:* prostaglandin D_2_; *PGE*_*2*_ *:* prostaglandin E_2_; *PGF*_*2α*_ *:* prostaglandin F_2α_; *PGI*_*2*_ *:* prostaglandin I_2_ or prostacyclin; *PI3K:* phosphatidylinositol 3-kinase; *PKA:* protein kinase A; *PKC:* protein kinase C; *PLCβ:* phospholipase Cβ; *PPARα:* peroxisome proliferator-activated receptor α; *PPARγ:* peroxisome proliferator-activated receptor γ; *Rap1:* Ras-related protein 1; *Rho:* Ras homologous; *rS6:* ribosomal protein S6; *RvD1:* resolvin D1; *RvD2:* resolvin D2; *RvD5:* resolvin D5; *RvE1:* resolvin E1; *STAT3:* signal transducer and activator of transcription 3; *TP:* thromboxane receptor; *TxA*_*2*_ *:* thromboxane A_2_; *12-HHT:* 12-hydroxyheptadecatrienoic acid; *11(S)-HpDPA*_*ω-6*_ *:* 11(S)-hydroperoxydocosapentaenoic acid; *12(S)-HETrE:* 12(S)-hydroxyeicosatrienoic acid; *12(S)-HETE:* 12(S)-hydroxyeicosatetraenoic acid; *14(S)-HpDPA*_*ω-6*_ *:* 14(S)-hydroperoxydocosapentaenoic acid; *20-HETE:* 20-hydroxyeicosatetraenoic acid.

The prostanoid receptors couple to a range of intracellular signaling pathways that mediate the effects of receptor activation in the cell (Table 1). While EP2, EP4, IP, and DP1 receptors activate adenylyl cyclase (AC) via G_αs_, increasing intracellular cyclic adenosine monophosphate (cAMP) and protein kinase A (PKA) activity, EP1, FP, and TP activate phosphatidylinositol metabolism via G_αq_, leading to the formation of inositol triphosphate (IP_3_) via the mobilization of intracellular free calcium (Ca^2+^) (Huang et al., 2004a). In addition to signaling through G_αq_, the FP and TP receptors couples to the small G-protein Rho via a G_α12/13_-dependent mechanism (Ricciotti and FitzGerald, 2011). EP3 isoforms can couple via G_αi_ or G_α12_ to elevate intracellular Ca^2+^, inhibit cAMP generation, and activate Rho (Ricciotti and FitzGerald, 2011). The DP2 couples to a G_αi_ to inhibit cAMP synthesis and increase intracellular Ca^2+^ (Schuligoi et al., 2010).

### 3.2 Leukotriene receptors

There are four known LT receptors subfamilies (Table 1). Two GPCRs are known to be associated with LTB_4_, leukotriene B_4_ receptor (BLT) BLT_1_ and BLT_2_. While BLT_1_ is known to be expressed on a number of blood cells including leukocytes (Yokomizo et al., 1997), eosinophils (Tager et al., 2000), cluster of differentiation (CD) 4^+^ and CD8^+^ effector T cells (Goodarzi et al., 2003; Tager et al., 2003), dendritic cells (Toda et al., 2010) and macrophages (Serezani et al., 2011), BLT_2_ is expressed ubiquitously in leukocytes, with high expression in mononuclear cells, such as CD8^+^ and CD4^+^ T-cells, and CD14^+^ monocytes (Toda et al., 2002). In leukocytes, BLT_1_ is coupled to the pertussis toxin-sensitive G protein (G_αi_) and its activation by LTB_4_ promotes Ca^2+^ mobilization, leukocyte chemotactic migration and lysosomal release (Goldman et al., 1985). In monocytes, both BLT_1_ and BLT_2_ have been reported to couple to G_αi_ to induce phosphorylation of mitogen-activated protein kinases (MAPKs) and PI3K/Akt (phosphatidylinositol 3-kinase/protein kinase B, Akt is also known as protein kinase B (PKB)), and nuclear factor-κB (NF-κB) activation (Sánchez-Galán et al., 2009). However, in human umbilical vein endothelial cells (HUVECs) LTB_4_ increases HUVEC adhesiveness for polymorphonuclear neutrophils (PMNs) through the increase of intracellular Ca^2+^, but it does not depend on pertussis toxin-sensitive G proteins (Palmblad et al., 1994). The BLT_2_ receptor is considered to be a receptor for several oxidized fatty acids, including 12-hydroxyheptadecatrienoic acid (12-HHT) and hydroxyeicosatetraenoic acids (HETEs) (Yokomizo et al., 2000) and in the blood vessel, BLT_2_ is expressed in endothelial cells (Yokomizo et al., 2018).

CysLTs regulate cell function through the cysteinyl leukotriene receptors CysLT_1_ and CysLT_2_ (Funk, 2001) (Table 1). CysLT_1_ is known as a high-affinity receptor for LTD_4_, whereas CysLT_2_ has similar affinity to LTC_4_ and LTD_4_ (Woszczek et al., 2007). Duah et al. (Duah et al., 2013) has demonstrated that while CysLT_1_ activation elicits proliferation of endothelial cells via extracellular signal-regulated kinase (ERK) phosphorylation, activation of CysLT_2_ increases intracellular Ca^2+^ and leads to endothelial cell contraction and barrier disruption via the Rho kinase pathway. Moreover, the *in vitro* activation of CysLT_1_ by LTD_4_ in monocyte/macrophage U937 cells produces second intracellular messengers through phospholipase Cβ (Crooke et al., 1989). LTD_4_ induces Ca^2+^ response via the pertussis toxin-sensitive G protein (G_αi/o_) in these cells (Pollock and Creba, 1990; Capra, 2004). In addition, LTC_4_ has been shown to activate CysLT_2_ in mouse platelets *ex vivo* to induce α-granule and TxA_2_ secretion (Capra et al., 2003; Cummings et al., 2013). In endothelial cells, CysLT_2_ couples to G_αq/11_ to activates PLCβ and IP_3_ signaling, and increase intracellular Ca^2+^ release, in response to interferon-γ (IFN-γ) stimulation *in vitro* (Woszczek et al., 2007).

### 3.3 Epoxyeicosatrienoic acid receptors

The CYP-derived epoxyeicosatrienoic acids (EETs) activates peroxisome proliferator-activated receptor γ (PPARγ) in endothelial cells in the presence of an epoxide hydrolase-specific inhibitor (Liu et al., 2005). Additionally, EETs inhibit the NF-κB activation and attenuate the NF-κB-dependent inflammatory responses by reducing cytokine-induced leukocyte adhesion to the vasculature (Node et al., 1999). Some vascular-related actions of the EETs include the activation of the signal transducer and activator of transcription 3 (STAT3) (Table 1). Specifically, 14,15-EET stimulates the tyrosine phosphorylation of STAT3 and its translocation from the cytoplasm to the nucleus to bind to vascular endothelial growth factor (VEGF) promoter in a Src-STAT3 activation signaling-dependent manner, which leads to VEGF expression and angiogenesis (Cheranov et al., 2008).

### 3.4 Hydroxyeicosanoid receptors

Hydroxyeicosanoids are known to activate cells through a number of mechanisms including activation of GPCRs. The ω-6-derived 12(S)-HETrE inhibits platelet function through selectively binding to the G_αs_-coupled prostacyclin receptor and activates a PKA-dependent signaling pathway (Tourdot et al., 2017) (Table 1). More recently, Cebo et al. has suggested that 12(S)-HETrE promotes C-X-C chemokine receptor type 7 (ACKR3, also known as CXCR7) ligation coordinated with IP to trigger the cAMP-PKA signaling pathway. Enhanced platelet expression of the chemokine receptor ACKR3/CXCR7 has been reported in coronary artery disease patients with reduced platelet aggregation (Cebo et al., 2022).

The eicosanoid 12(S)-HETE acts through binding to the G-coupled protein receptor 31 (GPR31) in platelets and human umbilical vein endothelial cells (HUVECs) (Van Doren et al., 2021) (Table 1). In platelets, 12(S)-HETE-GPR31 signals through G_αi_ to induce platelet activation and thrombosis. Activation of the GPR31 inhibits AC activity and results in Ras-related protein 1 (Rap1) and p38 activation (Van Doren et al., 2019). 20-HETE affects vascular function by binding to G-protein coupled receptor 75 (GPR75) coupled to G_αq/11_ in endothelial cells which results in PLC-IP_3_-mediated increases in intracellular Ca^2+^ (Garcia et al., 2017), activation of the Rho kinase (Randriamboavonjy et al., 2003) and the mitogen activated protein (MAP) kinase pathways (Muthalif et al., 2000) (Table 1). Additionally, studies have demonstrated that 20-HETE stimulates the production of inflammatory cytokines, including interleukin-8 (IL-8), IL-13, IL-4, and PGE_2_, in endothelial cells via activation of NF-kB and MAPK/ERK signaling pathways (Ishizuka et al., 2008), resulting in endothelial cell activation and endothelial dysfunction (Singh et al., 2007). In addition to regulation of the cells through activation of GPCRs, hydroxyeicosanoids, such as 11(S)-HpDPA_ω-6_ and 14(S)-HpDPA_ω-6_ selectively activate PPARα in platelets *ex vivo* which results in inhibition of PKC activity and reduction in Ca^2+^ mobilization (Yeung et al., 2020) (Table 1).

### 3.5 Specialized pro-resolving (lipid) mediator receptors

Recent studies have shown that the SPMs also exert their effects in the blood to regulate inflammation through GPCRs (Table 1). These receptors are typically able to interact with more than one SPM and conversely some SPMs are able to interact with several receptors, leading to some overlapping downstream signals and pathways. RvE1 binds to BLT_1_ on neutrophils (Arita et al., 2007), to the chemokine-like receptor 1 (ChemR23) and to the resolvin E1 receptor (ERV1) on monocyte/macrophages (Freire et al., 2017), platelets (Fredman et al., 2010), neutrophils (Chiang and Serhan, 2020), and VSMCs (Ho et al., 2010). The activation of BLT_1_ by RvE1 induces phosphorylation of the ribosomal protein S6 (rS6) in neutrophils (Freire et al., 2017), as well as RvE1 activation of ERV1/ChemR23, which results in phosphorylation of Akt and rS6 to enhance phagocytosis by human macrophages (Ohira et al., 2010). Additionally, treatment of HEK-ChemR23 cells with pertussis toxin inhibited RvE1-dependent ERK activation (Serhan et al., 2011b). Although it was shown in HEK cells, the pertussis toxin-sensitive G protein (Gα_i/o_)-dependent pathway has already been shown to be activated by LTD_4_ in macrophages, suggesting that ChemR23 might couple to a Gα_i/o_ to activate intracellular signaling in cells in the blood.

Regarding the D-series resolvins, while in human VSMCs RvD1 binds to the formyl peptide receptor 2 (ALX/FPR2) (also known as LXA_4_ receptor) (Ho et al., 2010), studies have suggested that RvD1 may interact with two GPCRs the ALX/FPR2 and the G-protein coupled receptor 32 (GPR32) in leukocytes and platelets (Krishnamoorthy et al., 2010; Lannan et al., 2017). Notably, lipoxins have been found to interact with the same receptors as RvD1, the ALX/FPR2 and GPR32 receptors (Chandrasekharan and Sharma-Walia, 2015). Recently, RvD2 was shown to bind to the G protein-coupled receptor 18 (GRP18) in leukocytes, including PMN, monocytes, and macrophages (Chiang et al., 2015). In macrophages, activation of GRP18 by RvD2 leads to cAMP release and phosphorylation of select kinases and transcription factors, such as cAMP-response element binding protein (CREB) and STAT3 (Chiang and Serhan, 2020). The RvD5 was described to activate the RvD1 receptor GPR32 in leukocytes and macrophages to reduce the expression of NF-κB (Chiang et al., 2012) (Table 1).

The MaR1 activates the leucine-rich repeat-containing G protein-coupled receptor 6 (LGR6) in neutrophils and macrophages/monocytes to increase the phosphorylation of CREB and ERK (Chiang et al., 2019; Chiang and Serhan, 2020). Moreover, studies have shown that MaR1 suppresses NF-κB activation in VSMC and vascular endothelial cells *in vitro* (Chatterjee et al., 2014; Akagi et al., 2015). PD1/NPD1 binds to the G protein-coupled receptor 37 (GPR37) to increase intracellular Ca^2+^ in macrophages (Chiang and Serhan, 2020) (Table 1).

## 4 Pro-inflammatory eicosanoids in the blood and the vessel

### 4.1 Prostaglandin E_2_

**FIGURE 3:**
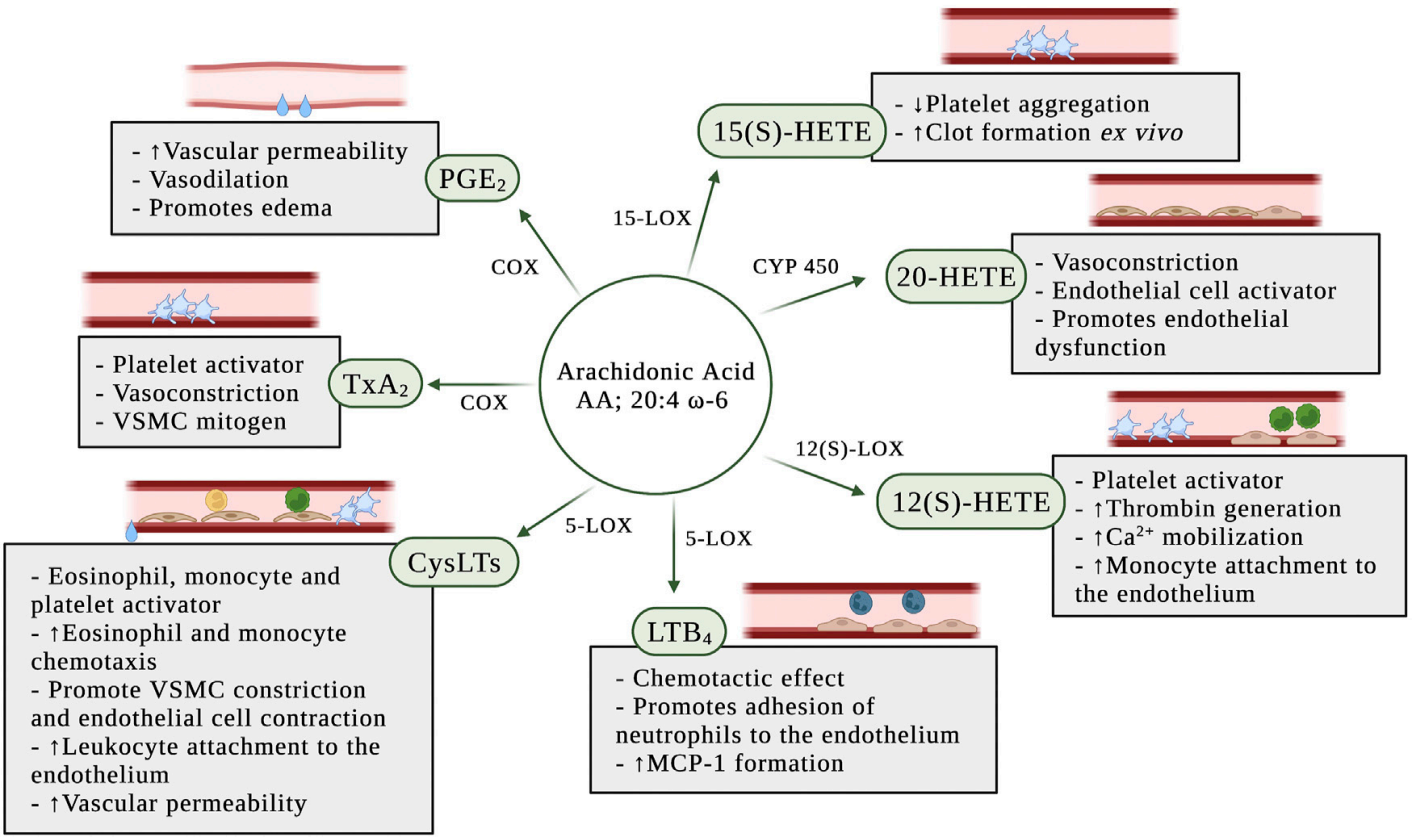
Pro-inflammatory effects of eicosanoids in the blood and the vascular wall. *AA:* arachidonic acid; *COX:* cyclooxygenase; *CYP450:* P450 cytochrome epoxygenase; *CysLTs:* cysteinyl leukotrienes; *LTB*_*4*_ *:* leukotriene B_4_; *MCP-1:* monocyte chemoattractant protein-1; *PGE*_*2*_ *:* prostaglandin E_2_; *TxA*_*2*_ *:* thromboxane A_2_; *VSMC:* vascular smooth muscle cell; *5-LOX:* 5-lipoxygenase; *12(S)-LOX:* 12(S)-lipoxygenase; *12(S)-HETE:* 12(S)-hydroxyeicosatetraenoic acid; *15-LOX:* 15-lipoxygenase; 1*5(S)-HETE:* 15(S)-hydroxyeicosatetraenoic acid; *20-HETE*: 20-hydroxyeicosatetraenoic acid.

PGs play a key role in the generation of the inflammatory response (Ricciotti and FitzGerald, 2011). They are ubiquitously produced and act as autocrine and paracrine lipid mediators to maintain local hemostasis in the body (Funk, 2001). While PG production is generally very low in uninflamed tissues, it increases immediately in acute inflammation before the recruitment of leukocytes and the infiltration of immune cells (Ricciotti and FitzGerald, 2011). In the blood vessel, one member of the PG family, PGE_2_, is synthesized mainly by platelets and macrophages (Cook, 2005). PGE_2_ has vasodilation effects and increases the permeability of postcapillary venules, early events in the inflammatory response (Funk, 2001) (Figure 3). Furthermore, PGs may synergize in the blood vessel with other pro-inflammatory mediators, such as histamine or bradykinin, to increase vascular permeability and promote edema (Funk, 2001; Khanapure et al., 2007).

### 4.2 Thromboxane A_2_

TxA_2_ is synthesized by macrophages and monocytes on the blood, and in large quantities by platelets (Cook, 2005; Ricciotti and FitzGerald, 2011; Yeung and Holinstat, 2011). Although TxA_2_ is an unstable compound with a half-life of 20–30 s (Cook, 2005), it has a wide range of effects on the blood vessel (Figure 3). TxA_2_ is a potent vasoconstrictor and VSMC mitogen. It is produced by aggregating platelets and acts as a direct platelet activator in addition to amplifying the platelet response to other platelet agonists (Praticò and Dogné, 2009).

### 4.3 Leukotrienes

#### 4.3.1 Cysteinyl leukotrienes

CysLTs are potent pro-inflammatory mediators produced during vascular injury (Colazzo et al., 2017). The cysLTs induce eosinophil and monocyte chemotaxis and activation (Funk, 2001), potentiate platelet activation (Yeung et al., 2017), promote vascular smooth muscle constriction and increase vascular permeability in post-capillary venules (Poeckel and Funk, 2010) (Figure 3). Duah et al. (Duah et al., 2013) demonstrated that LTC_4_ and LTD_4_ regulate endothelial cell function *in vitro* through the increase of endothelial contraction and induction of barrier disruption in the endothelial cell monolayer. In the same study, they also demonstrated that the cysLTs are able to promote attachment of leukocytes to the endothelial monolayer.

#### 4.3.2 Leukotriene B_4_

Although most attention has been focused on the COX-dependent pathway of the prostanoids’ biosynthesis, the 5-LOX-catalyzed oxygenation of AA play a role in inflammation through the formation of LTs (Poeckel and Funk, 2010). The 5-LOX pathway has long been recognized as a pro-inflammatory cascade and LTs are lipid mediators involved in inflammation and chemotaxis (Funk, 2001). Expression of 5-LOX is usually absent under normal physiologic conditions, but is induced by pro-inflammatory stimuli. Leukotriene B_4_ (LTB_4_) is a potent chemotactic effect on leukocytes (Yokomizo et al., 2018) and has been implicated in atherosclerosis (Bäck et al., 2005; Ketelhuth et al., 2015). *In vivo* and *in vitro* studies have shown that LTB_4_ promotes neutrophil chemotaxis, traffic and adhesion of monocytes to vascular endothelial cells (Friedrich et al., 2003), and increases the formation of monocyte chemoattractant protein-1 (MCP-1) (Huang et al., 2004b) (Figure 3).

### 4.4 Hydroxyeicosanoids

#### 4.4.1 *12(S)-Hydroxyeicosatetraenoic acid*

The early studies with 12(S)-hydroxyeicosatetraenoic acid (12(S)-HETE) had described anti-inflammatory, antiplatelet and anti-thrombotic effects (Fonlupt et al., 1991; Lagarde et al., 2018). However, most recent studies have shown that 12(S)-HETE potentiates platelet activation, thrombin generation, and calcium mobilization in the platelet (Yeung and Holinstat, 2011) (Figure 3). Furthermore, *in vitro* treatment of human aortic endothelial cells with 12(S)-HETE increased monocyte binding to endothelial cells (Patricia et al., 1999).

#### 4.4.2 20-Hydroxyeicosatetraenoic acid

The role of 20-hydroxyeicosatetraenoic acid (20-HETE) in the regulation of vascular tone and homeostasis promoting a prohypertensive response is due to its potent vasoactive effect (Miyata and Roman, 2005). 20-HETE causes vasoconstriction through its regulation of intracellular signaling (Muthalif et al., 2000) and membrane depolarization (Obara et al., 2002) in smooth muscle cells. Furthermore, studies have demonstrated that 20-HETE stimulates the production of inflammatory cytokines, including IL-8, IL-13, IL-4, and PGE_2_, in endothelial cells (Ishizuka et al., 2008) resulting in endothelial cell activation and endothelial dysfunction (Singh et al., 2007) (Figure 3).

## 5 Anti-inflammatory eicosanoids in the blood and the vessel

### 5.1 Prostaglandins

**FIGURE 4:**
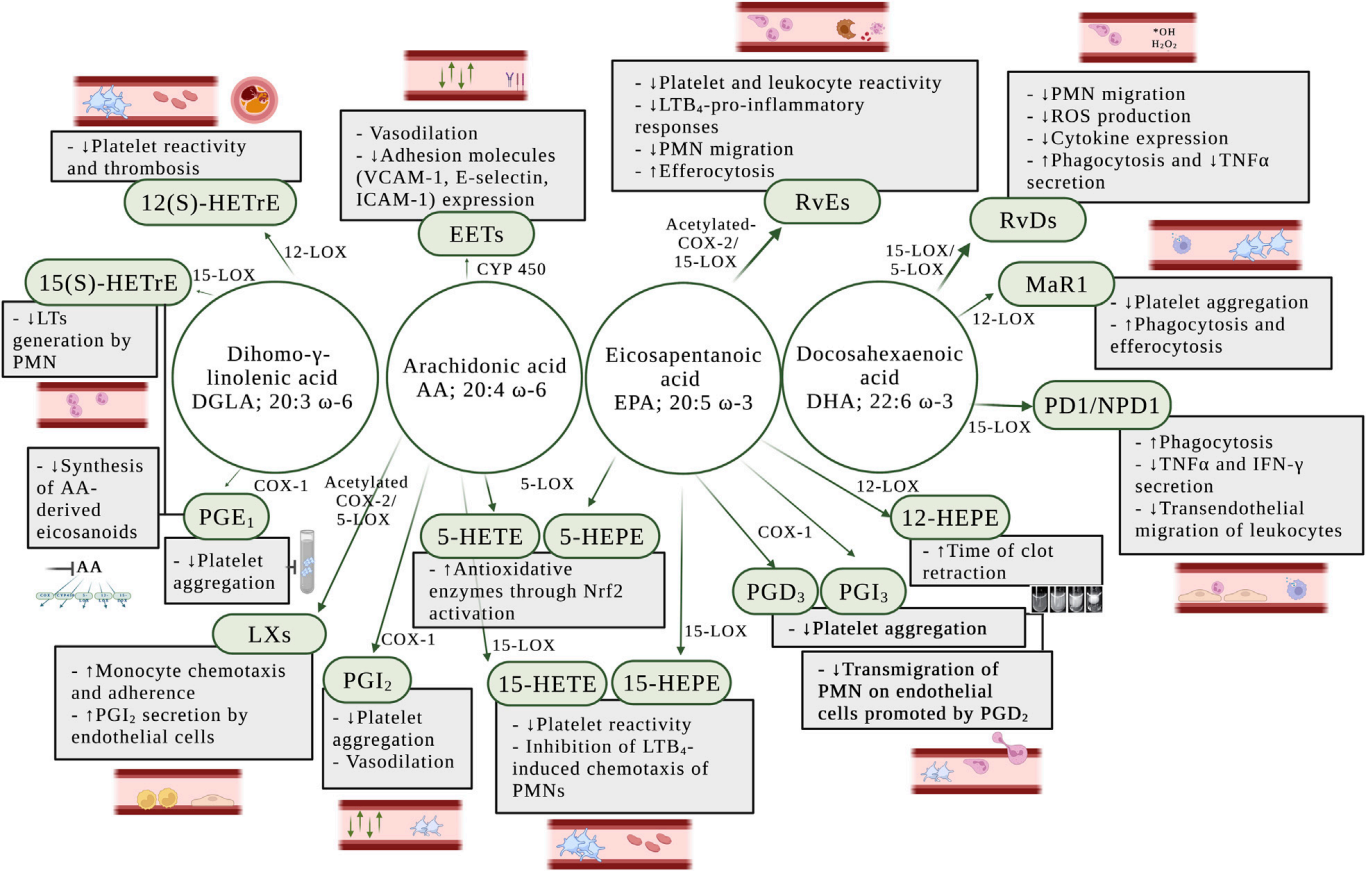
Anti-inflammatory effects of eicosanoids in the blood and the vessel wall. *AA:* arachidonic acid; *DGLA:* dihomo-γ-linolenic acid; *DHA:* docosahexaenoic acid; *EETs:* epoxyeicosatrienoic acids; *EPA:* eicosapentaenoic acid; *COX:* cyclooxygenase; *COX-1:* cyclooxygenase-1; *COX-2:* cyclooxygenase-2; *CYP450:* cytochrome epoxygenase; *ICAM-1:* intercellular adhesion molecule 1; *IFN-γ:* interferon-γ; *LTs:* leukotrienes; *LTB*_*4*_: leukotriene B_4_; *LXs:* lipoxins; *MaR1:* maresin 1; Nrf2: nuclear factor-erythroid factor 2-related factor 2; *PD1/NPD1:* protectin D1/neuroprotectin D1; *PGD*_*2*_: prostaglandin D_2_; *PGD*_*3*_: prostaglandin D_3_; *PGE*_*1*_: prostaglandin E_1_; *PGI*_*2*_: prostaglandin I_2_ or prostacyclin; *PGI*_*3*_: prostaglandin I_3_; *PMN:* polymorphonuclear leukocytes; *RvDs:* D-series resolvins; *RvEs:* E-series resolvins; *ROS:* reactive oxygen species; *TNFα:* tumor necrosis factor α; *VCAM-1:* vascular cell adhesion molecule 1; *12-HEPE*: 12-hydroxyeicosapentaenoic acid; *15-HEPE*: 15-hydroxyeicosapentaenoic acid; *5-HETE: 5*-hydroxyeicosatetraenoic acid; *5-LOX:* 5-lipoxygenase; *12-LOX:* 12-lipoxygenase; *12(S)-HETrE:* 12(S)-hydroxyeicosatrienoic acid; *15-LOX:* 15-lipoxygenase; *15-HEPE*: 15(S)-hydroxyeicosapentaenoic acid; *15-HETE: 15*(S)-hydroxyeicosatetraenoic acid; *15(S)-HETrE:* 15(S)-hydroxyeicosatrienoic acid.

Prostacyclin (PGI_2_) has been characterized to inhibit platelet aggregation and exerts vasodilator functions, as well as counterbalancing the actions of TxA_2_ (Schmid and Brüne, 2021) (Figure 4). It is produced primarily by vascular endothelial and VSMCs, but other cells such as fibroblasts and dendritic cells also synthesize PGI_2_ (Dorris and Peebles, 2012). PGI_2_ inhibits LPS-induced expression of pro-inflammatory cytokines in macrophages, dendritic cells, T cells and endothelial cells (Luttmann et al., 1996; Zhou et al., 2007a; Zhou et al., 2007b; Di Francesco et al., 2009). PGI_2_ can synergize with the anti-inflammatory cytokines IL-4 and IL-13 to selectively inhibit the release of pro-inflammatory cytokines from human peripheral mononuclear blood cells (Luttmann et al., 1999). Under inflammatory conditions including atherosclerosis, the production of PGI_2_ may increase, and this has been commonly considered a protective mechanism. Nonetheless, due to PGI_2_ acting mainly on TP receptors in vessels with limited IP receptor expression, an increase of its synthesis may lead to increased endothelium-derived vasoconstrictor activity (Luo et al., 2016).

Series 1 and series 3 PGs are well-known to inhibit platelet activity both *in vivo* and *in vitro* (Lagarde et al., 2013; Sergeant et al., 2016). PGE_1_ has been considered of biological interest as strong inhibitor of platelet function (Minno et al., 1979; Colman and Figures, 1984), whereas PGD_3_ effectively oppose the transmigration of neutrophils on endothelial cells promoted by PGD_2_ (Tull et al., 2009). Thus, PGD_3_ and PGI_3_ have been exhibited potent anti-aggregatory effect *in vitro* in human platelet experiments (Whitaker et al., 1979; Fischer and Weber, 1985) (Figure 4).

### 5.2 Specialized pro-resolving (lipid) mediators

#### 5.2.1 Lipoxins

The lipoxins A (LXA_4_) and B (LXB_4_) were two of the first SPMs to be identified and play a critical role in the down-regulation of acute inflammation and enhancement of resolution (Serhan, 2005). LXs increase monocyte chemotaxis and adherence, without causing degranulation or elevation of reactive oxygen species (ROS) (Scalia et al., 1997). It has been established that LXs regulate anti-inflammatory signaling in vascular homeostasis through the stimulation of PGI_2_ secretion by human endothelial cells (Brezinski et al., 1989) (Figure 4).

#### 5.2.2 D-series resolvins

Upon vascular injury, the resolvins D1 (RvD1) and D2 (RvD2) have been shown to regulate VSMC phenotypic response, including inhibition of proliferation, migration, monocyte adhesion, ROS production, and inflammatory cytokine expression (Miyahara et al., 2013) (Figure 4). Using mass spectrometry approaches, Cherpokova et al. (Cherpokova et al., 2019) has identified the kinetics of the formation of SPMs in the clot using a deep vein thrombosis animal model. In the same study, administration of RvD4 reduced thrombus burden, with less neutrophil infiltration and more pro-resolving monocytes in the clot. RvD5 promotes pro-resolving effects through enhancement of phagocytosis and reduction of expression of tumor necrosis factor α (TNFα) in neutrophils and macrophages (Chiang et al., 2012; Werz et al., 2018).

#### 5.2.3 E-series resolvins

The resolvin E1 (RvE1) was the first isolated and studied E-series resolvin and possesses anti-inflammatory and pro-resolving actions (Serhan and Petasis, 2011). In the blood, RvE1 has been shown to negatively regulate leukocytes *in vivo* and platelets *ex vivo* (Figure 4), by reducing U46619-, a TP receptor agonist, and ADP-stimulated platelet aggregation, and TxA_2_ generation (Dona et al., 2008), suggesting that RvE1 might inhibit P2Y_12_ receptor in platelets. Currently, P2Y_12_ receptor antagonists are used in association with aspirin as the most widely used antiplatelet therapy in cardiovascular diseases (Jackson and Schoenwaelder, 2003). In addition, RvE1 initiates resolution of inflammation through repolarization of human M1 macrophages toward resolution-type macrophages (Herová et al., 2015). Since RvE1 activates the BLT_1_ receptor in neutrophils, LTB_4_ action is inhibited which reduces LTB_4_-pro-inflammatory responses (Arita et al., 2007) (Figure 4).

RvE2 has also been reported to promote anti-inflammatory and pro-resolving effects through *ex vivo* inhibition of PMN chemotaxis and enhancement of nonphlogistic phagocytosis by macrophages (Oh et al., 2012). Recently, the inhibitory effect of RvE3 on neutrophil chemotaxis *in vitro* has been demonstrated (Isobe et al., 2012) and a new member of the EPA-derived resolvins E has been identified and termed resolvin E4 (RvE4). RvE4 is produced under physiologic hypoxia and has a resolving function. It stimulates human M2 macrophage efferocytosis of senescent erythrocytes and apoptotic neutrophils *in vitro* (Libreros et al., 2020) (Figure 4).

#### 5.2.4 Maresin 1

Maresin 1 (MaR1) plays a role in the resolution of inflammation by reducing platelet aggregation *ex vivo* (Freedman et al., 2020) and stimulating phagocytosis and efferocytosis in human and mouse phagocytes (Chiang and Serhan, 2020) (Figure 4).

#### 5.2.5 Protectin D1/Neuroprotectin D1

Studies have demonstrated that protectin D1/Neuroprotection D1 (PD1/NPD1) increases phagocytosis in macrophages, regulates TNFα and IFNγ secretion by activated T cells *in vitro* (Ariel et al., 2005), and limits transendothelial migration of leukocytes to prevent the infiltration of leukocytes into sites of inflammation (Serhan et al., 2015; Calder, 2020a; Chiang and Serhan, 2020) (Figure 4)**.** Moreover, synthetic PD1/NPD1 attenuated human PMN transmigration *in vivo* and *in vitro* in response to LTB_4_ and T cells (Serhan et al., 2015) **.**

Human leukocytes can form AT-(NPD1/PD1) via aspirin-acetylated COX-2 (Serhan et al., 2015). Studies have demonstrated that AT-(NPD1/PD1) has potent protective actions comparable to NPD1/PD1 *in vitro* and *in vivo*, reducing transendothelial PMN migration and enhancing efferocytosis of apoptotic human PMN by macrophages (Serhan et al., 2011a).

### 5.3 Hydroxyeicosanoids

#### 5.3.1 5-Hydroxyeicosanoids

In neutrophils, EPA and AA are metabolized by 5-LOX to form 5-hydroxyeicosatetraenoic acid (5-HEPE) and 5-hydroxyeicosatetraenoic acid (5-HETE), respectively. Both 5-HEPE and 5-HETE have been shown to induce antioxidative enzymes in vascular endothelial cells through activation of a nuclear factor-erythroid factor 2-related factor 2 (Nrf2)-dependent mechanism through their metabolites, 5-oxo-EPE and 5-oxo-HETE (Nagahora et al., 2017) (Figure 4).

#### 5.3.2 12(S)-Hydroxyeicosanoids

The 12(S)-hydroxyeicosapentaenoic acid (12(S)-HEPE) is the eicosanoid formed through oxygenation of EPA by 12(S)-LOX. Pre-treatment of whole blood with EPA prolonged the time of clot retraction *ex vivo* (Figure 4), suggesting that EPA-derived eicosanoids, such as 12(S)-HEPE, might regulate blood clotting and play a role in clot resolution (Ikei et al., 2012).

DGLA is an ω-6 fatty acid that is oxidized in the platelet by 12(S)-LOX to form 12(*S*)-HETrE. This metabolite has been shown to attenuate platelet activity and thrombosis (Ikei et al., 2012). The antiplatelet role of 12-HETrE was determined by demonstrating its ability to inhibit platelet aggregation *in vitro* and attenuate clot formation *in vivo* (Figure 4) through selective activation of the prostacyclin receptor in platelets (Yeung et al., 2016; Tourdot et al., 2017; Yeung and Holinstat, 2017).

#### 5.3.3 15-Hydroxyeicosanoids

The 15-LOX-derived eicosanoids from AA, DGLA, and EPA are 15(S)-hydroxyeicosatetraenoic acid (15(S)-HETE), 15-hydroxyeicosatrienoic acid (15(S)-HETrE), and 15(S)-hydroxyeicosapentaenoic acid (15(S)-HEPE), respectively. While 15(S)-HETrE, 15(S)-HETE and 15(S)-HEPE have been shown to inhibit platelet reactivity (Guichardant et al., 1988; Vanderhoek et al., 1991) (Figure 4), other studies have observed a pro-aggregatory effect of 15(S)-HETE on platelet function (Setty and Stuart, 1986; Vijil et al., 2014) and an increase of clot formation in human whole blood pre-treated *ex vivo* with 15(S)-HETE (Lundqvist et al., 2016) (Figure 3).

Moreover, studies have suggested that DGLA inhibits the synthesis *in vitro* of LTB_4_ in neutrophils through the formation of 15(S)-HETrE (Iversen et al., 1991; Chilton-Lopez et al., 1996) (Figure 4). Additionally, 15(S)-HETE has been shown to inhibit LTB_4_-induced chemotaxis of PMNs *in vitro* (Ternowitz et al., 1988; Takata et al., 1994).

### 5.4 Epoxyeicosatrienoic acids

Epoxyeicosatrienoic acids (EETs) are generated from AA by CYP450 enzymes and promote the active termination of inflammation by a broad array of anti-inflammatory and pro-resolving actions. EETs were found to have direct effects on the large-conductance Ca^2+^-activated potassium (K^+^) channels in vascular smooth muscle cells (Campbell and Harder, 1999). This mechanism contributes to the effect of EETs as endothelium-derived hyperpolarizing factor to hyperpolarize and relax arterial smooth muscle (Li and Campbell, 1997). EETs present functional relevance in vascular inflammation primarily due to their role in the reduction of vascular cell adhesion molecule 1 (VCAM-1), E-selectin and intercellular adhesion molecule 1 (ICAM-1) expression, and prevention of leukocyte adhesion to the vascular wall (Node et al., 1999) (Figure 4).

## 6 Discussion

Polyunsaturated fatty acids and their bioactive eicosanoids play a critical role in human health and diseases through regulating inflammation in the blood and the vessel. The role of the ω-6 PUFA AA in inflammation through formation of eicosanoids is well established. While the AA-derived eicosanoids, including PGE_2_, TxA_2_ and LTs, are well-known as pro-inflammatory mediators in the blood, the COX-derived PGs PGI_2_ from AA, and the PGs series 1 and 3 from EPA and DGLA, respectively, have a critical role in counterbalancing pro-inflammatory states to attenuate inflammation in the blood and the vascular wall. More recently, studies in the eicosanoid-inflammation field have additionally identified a wide class of bioactive metabolites and SPMs derived from AA, DGLA, EPA, and DHA. As the classic eicosanoids (PGs, LTs and Txs), the bioactive metabolites have pro- or anti-inflammatory effects, whereas the SPMs are currently extensively studied due to their effects on the attenuation of pro-inflammatory eicosanoid actions and active contribution to the resolution of inflammatory tissue. This review has outlined the function of COX-, LOX- and CYP 450-derived eicosanoids from PUFAs and elucidated their mechanistic regulation of the inflammation process in the blood and the vessel.

As a result of their widespread expression in the blood and the vascular wall, eicosanoids and their metabolites are involved in the pathogenesis and the development of inflammatory diseases such as atherosclerosis, hypertension, diabetes mellitus and more recently, COVID-19. As an example, alterations in the formation of bioactive metabolites, such as 20-HETE, have been reported in inflammatory diseases such as hypertension, diabetes (Miyata and Roman, 2005) and cardiovascular disease (Zu et al., 2016). Due to their critical involvement in eicosanoid biosynthesis, alterations in the expression of oxygenases also play a role in the pathogenesis of inflammatory diseases such as atherosclerosis and diabetes. While the upregulation of 5-LOX expression, leading to production of Cys-LTs and LTB_4_, has been reported at the site of atherosclerotic plaques (Whatling et al., 2007; Riccioni et al., 2010), increased 12-LOX activity or expression has been implicated in the functional loss of insulin secretion or production in beta-cells of the pancreatic islets, which may impair blood glucose regulation leading to the development of diabetes (Ma et al., 2010)**.** Synthesis of PGE_2_ has also been suggested to be up-regulated in atherosclerosis. Using an animal model of atherosclerosis, Gross et al. have shown that PGE_2_ is produced in the arterial wall in response to inflammation and is detected in atherosclerotic plaques. In addition, the authors have demonstrated that PGE_2_ enhances atherothrombosis *in vivo* (Gross et al., 2007). Increased production of PGE_2_ was recently identified in the blood of COVID-19 patients. These patients were found to have higher PGE_2_ levels which were correlated positively with the severity of the disease (Ricke-Hoch et al., 2021). Coronavirus infection activates endoplasmic reticulum stress signaling, which, in turn, can induce the biosynthesis of PGE_2_ (Chopra et al., 2019). Thus, an increased level of PGE_2_ may be involved in the hyperinflammatory response in COVID 19 infection (Hammock et al., 2020).

Due to their effects on promoting inflammation, the eicosanoids are potential targets for the treatment of these diseases, as well as the enzymes and receptors implicated in their formation. For example, the inhibition of 12-LOX in platelets, using the pharmacological inhibitor ML355, reduces platelet aggregation *ex vivo* and impairs clot formation *in vivo* (Adili et al., 2017). The prostacyclin analogs, such as iloprost and selexipag, are used to treat pulmonary arterial hypertension due to their vasodilatory and anti-platelet effects through activation of the prostacyclin receptor (Sitbon et al., 2015; Mandras et al., 2021). The inhibition of pro-aggregatory effects of TxA_2_ through acetylation of COX-1 in platelets is the pharmacological basis for aspirin, used in association with a P2Y_12_ receptor antagonist in dual antiplatelet therapy to treat cardiovascular diseases and prevent the recurrence of major cardiovascular events due to thrombosis (Schrör and Rauch, 2015). The role of TxA_2_ in the impairing endothelial function is highly associated with pathogenesis of atherosclerosis. Studies have shown that mice deficient in TP and IP demonstrated an accelerated atherogenesis in the blood vessel (Kobayashi et al., 2004). Notably, the acetylation of COX by aspirin can trigger alternative biosynthesis pathways forming bioactive metabolites and SPMs (Figure 2) which might provide additional anti-inflammatory effects promoted by aspirin treatment.

Studies have shown that increased intake of ω-3 PUFAs (EPA and DHA) results in increased amounts of these fatty acids in blood lipids, leukocytes and platelets (Browning et al., 2012). The increased level of ω-3 PUFAs in leukocytes and platelets has been demonstrated to result in a reduction of the capacity of these cells to produce pro-inflammatory eicosanoids from AA, such as PGs and LTs (Calder, 2020b), and to regulate the function of these cells by attenuating platelet reactivity and increasing leukocyte response to inflammation (Faber et al., 2011; Yamaguchi et al., 2022). Notably, the concentration of several bioactive metabolites, including hydroxy- and epoxyeicosanoids derived from AA, EPA and DHA, were increased in the plasma of normo- and hyperlipidemic patients following supplementation with EPA and DHA (Schuchardt et al., 2014; Schmöcker et al., 2018). Moreover, studies have detected higher levels of the SPMs, such as RvD1 and RvD2, in the plasma and serum of individuals with an increased intake of EPA and DHA (Calder, 2020a). Thus, given the evidence of diverse supplementary studies, modulating the levels of PUFAs mediated by ingestion or supplementation might provide beneficial effects in attenuating the inflammation process in the blood and the vessel.

The SPMs have been recently described as positive modulators on resolution and termination of inflammation. Studies have indicated that RvE1 might control vascular inflammation in atherosclerosis. RvE1 has been shown to protect against atherogenesis in an animal model of atherosclerosis (Hasturk et al., 2015) and Laguna-Fernandez et al. (Laguna-Fernandez et al., 2018) have demonstrated that targeted deletion of the RvE1 receptor ERV1/Chem23 in a hyperlipidemic animal model was associated with proatherogenic signaling in macrophages, increased oxidized low-density lipoprotein uptake, reduced phagocytosis, and increased atherosclerotic plaque size and necrotic core formation, suggesting that RvE1 might have protective effects during atheroprogression (Salic et al., 2016). Additionally, the administration of the D-series resolvin RvD4 to mice of a deep vein thrombosis model has been shown to reduce thrombus formation and improve clot resolution (Cherpokova et al., 2019), suggesting that the delivery of SPMs might help to regulate thrombosis and inflammation in cardiovascular diseases. Thus, SPMs may be considered as potential therapeutic approaches for prevention or resolution of inflammation or insult in the vessel.

The discovery of SPMs was first reported in exudates (Serhan et al., 2011b) and the investigation of the effects of SPMs on the blood and the vessel is currently in early stages. Studies using *in vitro* assays and animal models have described the SPMs’ ability to contribute to resolution of inflammation through regulation of cell function in the blood and the vessel (see review (Chiang and Serhan, 2020)), but the physiological relevance of these effects depends on the endogenous concentration of SPM *in vivo*. The biosynthesis of SPMs has been characterized using *in vitro* studies (Isobe et al., 2012; Libreros et al., 2020; Perry et al., 2020) and other studies have demonstrated the ability of blood cells such as neutrophils and macrophages to form SPMs *in vitro* (Werz et al., 2018; Mainka et al., 2022). In addition, despite several studies having detected SPMs in human samples including plasma and serum (see review (Calder, 2020a)), the concentration of SPMs was at low levels (picogram/picomolar to nanogram/nanomolar range) (Mainka et al., 2022; Schebb et al., 2022) and the analysis of low concentrations of low SPMs can be an analytical challenge and it may affect the detection and quantification process of these metabolites in the sample. Indeed, there is a current controversy in the field based on differences in the methodology and analytical instrumentation used to detect the SPMs in biological samples (Schebb et al., 2022), which demonstrates that a deeper investigation is warranted to provide a better understanding of the concentration range of SPMs circulating in the human bloodstream and whether SPMs at these concentrations are able to regulate resolution of inflammation in the blood and the vessel.

The studies using *in vitro* and *in vivo* approaches in cellular and animal models, and the analysis of samples collected from humans, have significantly contributed to the current understanding of the mechanistic regulation of eicosanoids in inflammation. It resulted in a large body of evidence about the role of the classical pro- and anti-inflammatory eicosanoids derived from the 20-carbon PUFAs AA, DGLA and EPA, in inflammation in the blood. However, a better understanding of the mechanistic regulatory effects of the most recently discovered eicosanoids, including SPMs and bioactive metabolites, in the regulation of inflammatory states and their contribution to the resolution of inflammation in the blood and the vascular wall is warranted. Furthermore, it is important to highlight that, although there is evidence of the synthesis of SPMs by cells in the blood, whether the biosynthesis of some SPMs occurs in the blood and the biological relevance of this process still need to be further elucidate. Hence, the role of eicosanoids in inflammation in the blood and the vessel is currently a focus of much research in the inflammation field which might help to position the anti-inflammatory bioactive eicosanoids as a novel therapeutic approach to treat inflammatory diseases that affects the blood and the vascular wall.
